# Histo-blood group antigens as divergent factors of groups A and C rotaviruses circulating in humans and different animal species

**DOI:** 10.1080/22221751.2020.1782270

**Published:** 2020-07-14

**Authors:** Dandan Zhao, Yang Liu, Pengwei Huang, Ming Xia, Weiwei Li, Ming Tan, XuFu Zhang, Xi Jiang

**Affiliations:** a School of Traditional Chinese Medicine, Southern Medical University, Guangdong, People’s Republic of China; b Tianjin Key Laboratory of Molecular Nuclear Medicine, Institute of Radiation Medicine, Chinese Academy of Medical Sciences and Peking Union Medical College, Tianjin, People’s Republic of China; c Division of Infectious Diseases, Cincinnati Children’s Hospital Medical Center, Cincinnati, OH, USA; d Department of pediatrics, University of Cincinnati College of Medicine, Cincinnati, OH, USA

**Keywords:** Rotavirus, HBGA, evolution, receptor, diarrhea

## Abstract

Histo-blood group antigens (HBGAs) have been found to be important host susceptibility factors or receptors for human rotavirus (RVs) with genotype-specific host ranges, impacting the disease patterns, epidemiology, and strategy development against RV diseases in humans. However, how the glycan factors contribute to RV diversity and host ranges to different animal species remains unclear. In this study using recombinant VP8* proteins as probes to perform glycan array analyses of RVs, we observed a wide range of glycan-binding profiles, including those binding to sialic acid-containing glycans, among group A (RVA) and group C (RVC) RVs that mainly infect different animal species. A tri-saccharide glycan Galα1-3Galβ1-4Glc containing a terminal α-Gal was recognised by multiple RVA/RVC genotypes, providing valuable information on RV evolution under selection of the step-wisely synthesised HBGAs in many animals before they were introduced to humans to be human pathogens. Saliva binding studies of VP8* also revealed strain-specific host ranges or species barriers between humans and these animal RV genotypes, further improved our understanding on RV host ranges, disease burdens, epidemiology, and vaccine strategy against RVs.

## Introduction

Rotaviruses (RVs) are an important cause of severe diarrhea in young children with high morbidity and mortality, claiming approximately 200,000 lives each year worldwide [1]. They are double-stranded RNA viruses, consisting 5 serological species (A–E) and two additional tentative species (F and G) [2]. RVs belonging to species A, B and C (RVA, RVB and RVC) are known to infect humans and various animals, whereas RVs of species D, E, F and G (RVD,RVE, RVF and RVG) have only been identified in animals, mostly birds [2]. RVs contain an 11-segmented dsRNA genome that encode 6 structural proteins and 6 nonstructural proteins. The structural proteins VP4 and VP7 constitute the outermost layer of the triple layer RV capsid. Based on the sequences of VP4 and VP7 proteins, RVs are divided into different P (VP4) and G (VP7) genotypes, respectively. The spike protein formed by VP4 can be cleaved into two fragments, including VP5* corresponding to the basis and VP8* that forms the distal head of the spike protein. The VP8* interacts with the glycan receptors on the host cell surface to initiate RV infection, while VP5* facilitates penetration of RVs into host cells [3–5].

Early studies showed that some animal RVAs recognise the terminal sialic acids (SAs) as host receptors for attachment and these RV strains are referred as SA-dependent based on neuraminidase-sensitive assays on RV replication in cell cultures [6,7]. However, most human RVAs were found neuraminidase-insensitive and thus are referred as SA-independent [6]. Recent discoveries that major human RVs recognise HBGA as receptors or host susceptible factors for RV attachment to host cells support this notion [8–12].

HBGAs are complex carbohydrates consisting of three major families, the ABH(O), secretor, and Lewis families that are high diverse and distribute differently in the world population. HBGAs are synthesised step-wisely by glycosyl transferases that are specific for individual ABH and Lewis families [13]. The biosynthetic processes are genetically controlled, resulting in polymorphic HBGA types, which is evolutionarily regulated in humans and some animal species. Significant advancements have been made in understanding evolution of major human RVs following step wise adaptations from an animal host origin to humans under selection of strain-specific HBGA glycans, reflecting the step-wisely synthesised HBGAs in humans[8,14–16]. This new knowledge provides valuable information on disease burdens and epidemiology, as well as vaccine development against RVs. However, how the diverse HBGAs are involved in host ranges and evolution of RVs among different animal species before these RVs become human pathogens infecting different human populations remains unknown.

In this study, we performed *in vitro* binding studies using recombinant VP8* proteins of the RVA P[I] genogroup that mainly infect animals to explore the roles of HBGAs in binding and host ranges to different animal and human hosts. A wide range of binding profiles to different HBGA glycans was found among strains representing different genotypes infecting humans and different animal species. Comparison of the binding profiles identified a potential evolutionary lineage from a potential ancestor with an animal host origin under selection by the step-wisely synthesised HBGAs in humans and many animal species. These findings significantly improved our understanding on RV host ranges and epidemiology. We also performed similar studies of RVCs and found the same principles of RV evolution and host ranges that may apply to RVCs and other RV groups/species.

## Material and methods

Phylogenetic analysis of the VP8* sequence. The RV VP8* amino acid sequences from thirty-eight RVA and three RVC contained the intact protease-resistant VP8* core (amino acid (aa) 46–231)from the GenBank were used to perform alignment by the Laser gene software followed to build phylogenetic trees by the MEGA, versopm 4.1, software. A reference VP8* sequence from an RVB was included in the phylogenetic trees to estimate genetic distance among RV groups.

Expression and purification of VP8* proteins in *E. Coli.* The VP8* sequences (amino acids 46-231) from different RVAs and RVCs(EDIM: AF039219, EMcN: AY267006, WC3: GU565077, P[5]:GQ496266, P[7]:QAY29504.1, P[9]: AB077766,P[10]:EU791922, P[14]: GQ398013, P[15]:JQ013506,P[19]: DQ887060, P[23]:QAY29526.1, P[25]: GU199495,P[28]: EU805773.1, P[30]:EU486960) were expressed in *E. coli* BL21 and purified by GST-tag affinity purification, as described previously [9].

Glycan array analyses of recombinant VP8* proteins. Initial ligand screenings for different RVA and RVC VP8* were performed by the Protein-Glycan Interaction Core of CFG (Consortium for Functional Glycomics) (The glycan library information is available from the website http://www.functionalglycomics.org/ ). The recombinant GST-VP8* proteins were applied to individual glycan arrays at a protein concentration of 50 and 200 µg/ml and the bound GST-VP8* were detected using a fluorescent-labeled anti-GST monoclonal antibody. Relative fluorescent units (RFU) of each glycan were calculated to rank the reactivity in the interaction with individual RV VP8*s.

Binding of recombinant RV VP8* to human saliva. A set of previously characterised adult saliva samples with known ABO, secretor, and Lewis phenotypes were used in binding assays with the GST-VP8* fusion proteins [9].

## Results

The VP8* sequences of RVAs are genetically segregated from RVCs. 48 VP8* reference sequences representing 35 P genotypes in five genogroups (P[I] – P[V]) of RVAs [8] plus three new genotypes (P[36], P[37] and P[38]) [17–20] were analysed. The newly identified genotypes P[36] and P[37] were sorted into P[I] genogroup and the P[38] genotype into the P[V] genogroup. Genogroup P[I] contains the most P genotypes (25 genotypes) including P[1]-P[3], and P[7] that are known as sialic acid-dependent RVs, infecting monkeys, dogs, bovine, porcine, horses and occasionally humans, as well as sialic acid-independent RVs that infect a wide ranges of animal species, including bovine, porcine, equine, monkey, lamps, murine, rabbits and humans. P[36] infects sugar glider [19], while P[37] infects pheasant [17,18].

Significant advancements on the P[II] RV evolution from an ancestor in P[I] genogroup following a step wise evolution with an animal host origin and adaptation to humans and become the major human pathogens have been made [8,14,15]. P[III] contains three genotypes (P[9],P[14] and P[25]) that recognise the type A antigens and infect humans, cats and bovine with the A antigen as a potential cross-species transmission factor between humans and these animal species [8]. P[IV] has one genotype P[11] that recognises the type 2 HBGAs and can switch binding to type 1 HBGAs and infect bovines and humans. The P[V] genogroup included four P genotypes (P[17], P[30], P[31] and P[35]) that mainly infect avian and bovine species plus the new P[38] genotype that infects Turkey [20], the host receptor ligand recognised by P[V] RVs remain unknown.

**Figure 1.**
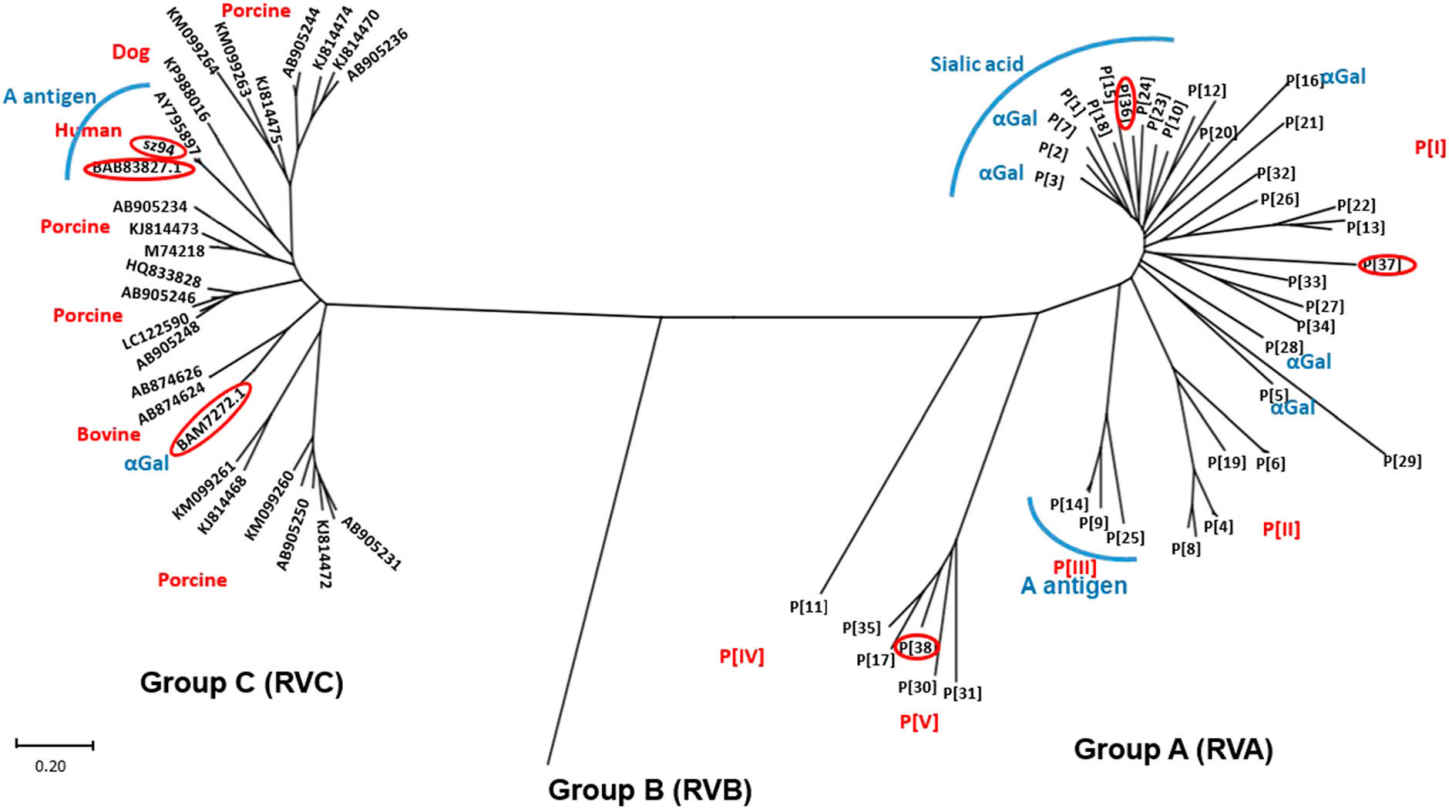
Phylogeny of group A rotaviruses. The group A RVs have been divided into 38 P genotypes (P[1] – P[38]) including 3 new genotypes (P[36]-P[38]) based on the major surface spike protein VP4/VP8* based on previous study [8]. A phylogeny dividing 38group A RV genotypes into in five P genogroups (P[I] – P[IV]) is shown. A total of 34 VP8* amino acid (aa) sequences (aa 46 to 231) of RVCs from GenBank have been grouped into multiple porcine and one for each of bovine and human genogroups, consisting with genetic grouping results reported by Sun et al. [21].

We also performed phylogenetic analyses of VP8* sequences for RVCs for a comparison with that of RVAs. A total of 32 VP8* sequences (aa 46–231) of RVCs from GenBank have been grouped into multiple porcine RV lineages, as well as several other lineages, each for bovine, dog and human genogroups (Figure 1), which is similar to the genetic grouping results reported by Sun et al. [21]. The VP8* sequences of RVCs were segregated from those of RVAs in the phylogenetic tree (Figure 1), consistent with the VP6-based classification of RVA and RVC as distinct groups/species.

**A trisaccharide glycan Galα1-3Galβ1-4Glc containing a terminal αGal is recognised by multiple RVA and RVC genotypes.** To understand the diversity and evolution of major animal RVs, we performed glycan microarray analyses on selected RVs representing multiple RVA genotypes infecting different animal species and two RVC genotypes that infect human and bovine, respectively. Widely variable glycan binding profiles with top list glycans have been detected for individual genotypes. Interestingly, a trisaccharide glycan Galα1-3Galβ1-4Glc (glycan number 117) containing an αGal was found to be recognised by multiple strains representing one RVC and six RVA genotypes, indicating evolutionary connections among these genotypes with the αGal glycan as a common receptor (Figure 1). Four strains exhibited strongest binding signals to the αGal containing glycans, including two murine RVAs in the P[16] genotype (the prototype murine RV EDIM and a variant murine RV EMcN [22,23]), P[3] genotype in RVA that infects animals and humans, and a bovine strain in a RVC genotype. It is known that the αGal transferase responsible for synthesis of αGal glycan is produced in some animal species, but the gene encoding the specific αGal transferase is inactive in humans [24]. Thus, these RVA and RVC genotypes may have a common ancestor with an animal host origin, e.g. mouse and bovine for RVAs and RVCs, respectively.

**Figure 2.**
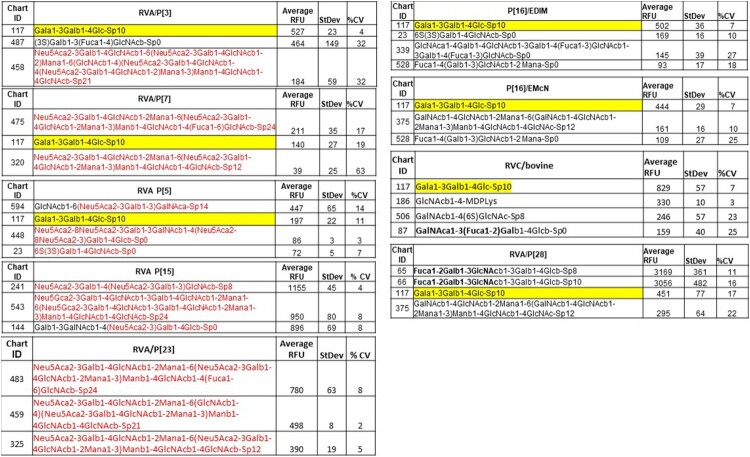
Glycan array results of selected RV strains representing different RVA and RVC genotypes. The top list glycans from an array library contains 610 glycans recognised by each RV strain representing different RVA and RVC genotypes are shown. The sialic acid-containing glycans are highlighted red for individual strains. The αGal trisaccharide was highlighted in yellow. All nine strains gave perfect matched relative fluorescent unites (RFUs) of αGal vs. other top-list glycans between two concentrations (50 and 5 µg) of VP8* proteins tested for each strain except two strains (P[16]/EMcN and RVA/P[5]) that had one glycan position changes at the 3rd and/or 4th glycans in the top-lists, respectively, but the ranks of αGal did not change (data not shown).

**Sialic acids are important host ligands for many animal RVAs.** RVAs representing two P[I] genotypes (P[3] and P[7]) known as sialic acid (SA)-dependent and another two P[I] genotypes known as SA-independent (P[15] and P[23]) [7,25,26] have been found binding SA-glycans at high affinity (Figure 2). Interestingly, these four SA-binding genotypes are genetically closely related and form a cluster together with other four SA-dependent genotypes (P[1], P[2], P[16] and P[20], excepting P[18], Figure 1 and Table 1) in the RVA P[I] genogroup. These findings indicated a new direction of RV evolution under selection of SA-containing glycans, consistent with the previous finding that many animal RVAs are SA-dependent [6,7].

**Table 1.** Summary of in vitro binding results of selected group A rotaviruses.

Genotype	Genogroup	Host species	SA-depend	Glycans arrays	Mucin arrays	Saliva binding
P[1]	P[I]	Bovine/Huan/porcine	Yes	ND	ND	ND
P[2]	P[I]	Simian/Human	Yes	ND	ND	ND
P[3]	P[I]	Canine/Human/Feline/simian//bovine/caprine	Yes	ND	ND	+
P[5]	P[I]	Bovine	No	ND	+	+
P[7]	P[I]	Porcine/Bovine/Human	Yes	SA+	+	+
P[10]	P[I]	Human	No	SA-	+	+
P[13]	P[I]	Porcine	No	SA-	ND	ND
P[15]	P[I]	Lamb/Bovine	No	SA+	+	+
P[16]	P[I]	Murine (EDIM)	Yes	ND	ND	+
P[16]	P[I]	Murine (EMcN)	Yes	ND	ND	+
P[18]	P[I]	Equine	No	ND	ND	ND
P[20]	P[I]	Bovine	Yes	ND	ND	ND
P[23]	P[I]	Porcine	No	SA+	ND	ND
P[28]	P[I]	Human	Unknown	SA-	ND	+
P[36]	P[I]	Sugar Glider	Unknown	ND	ND	ND
P[37]	P[I]	Pheasant	Unknown	ND	ND	ND
P[6]	P[II]	bovine	unknown	SA-	ND	+
P[19]	P[II]	Porcine/Human	No	SA-	ND	+
P[9]	P[III]	Human/Feline/Canine	No	ND	+	+
P[14]	P[III]	Human/Bovine/Lupine	Yes	ND	+	+
P[25]	P[III]	Human	No	SA-	ND	+
P[30]	P[V]	Avian	No	SA-	ND	ND
P[38]	P[V]	Turkey	Unknown	ND	ND	ND

Interestingly, a step-wise adaptation with a switch from an αGal- to an SA-binding mode has been deduced among five SA-dependent genotypes in the order of P[3]→P[7] →(P[5]/P[15]/P[23]) (Figure 2) by analysing their glycan binding profiles. For example, the P[3] RVAs recognised αGal as the top-list glycan at high affinity with a 3rd list SA-glycan at a low affinity (Figure 2), indicating that they are at an early stage of RV evolution that still relies on the αGal glycan as their host receptor. In addition, P[3] RVAs also had a comparable high binding affinity to the 2^nd^ list glycan [(3S)Galb1-3(Fuca1-4)GlcNAcb] with the backbone of the type 1 HBGA precursor, suggesting an intermediate stage of divergence with a possible flexible binding interface to both αGal- and SA-epitopes, possibly responsible for their broad host ranges to many animal species (canine, feline, simian, bovine and caprine) and humans [8] . In the case of P[7], however, αGal was ranked 2^nd^ with a comparable binding affinity to that of the No. 1 SA-containing glycans (Figure 2), therefore represents a later stage of divergence with a narrower host ranges, infecting porcine, bovine and humans [8]. Finally, P[15], P[5], and P[23] seemed have further shifted to bind SA-containing glycans, explaining their narrow host ranges to lamb and bovine, bovine, and porcine only, respectively. The dual recognition of αGal and SA-glycans by the P[5] RVs has been demonstrated by Alfajaro et al., by studying the bovine RV G6P[5] WC3 and its mono-reassortant G4P[5] RotaTeq vaccine strains [27].

**Animal RVAs also recognise a wide range of non-sialic acid glycans.** P[28] is the only genotype characterised in this study in the RVA P[I] genogroup that infects humans exclusively. Glycan array analyses showed that the P[28] VP8* recognised the H type 1 HBGA precursors (Figure 2), a property very similar to that of the P[6] and P[19] human strains in RVA P[II] genogroup, explaining why P[28] also causes sporadic cases in humans similar to that of P[19] and P[6][14].

**Figure 3.**
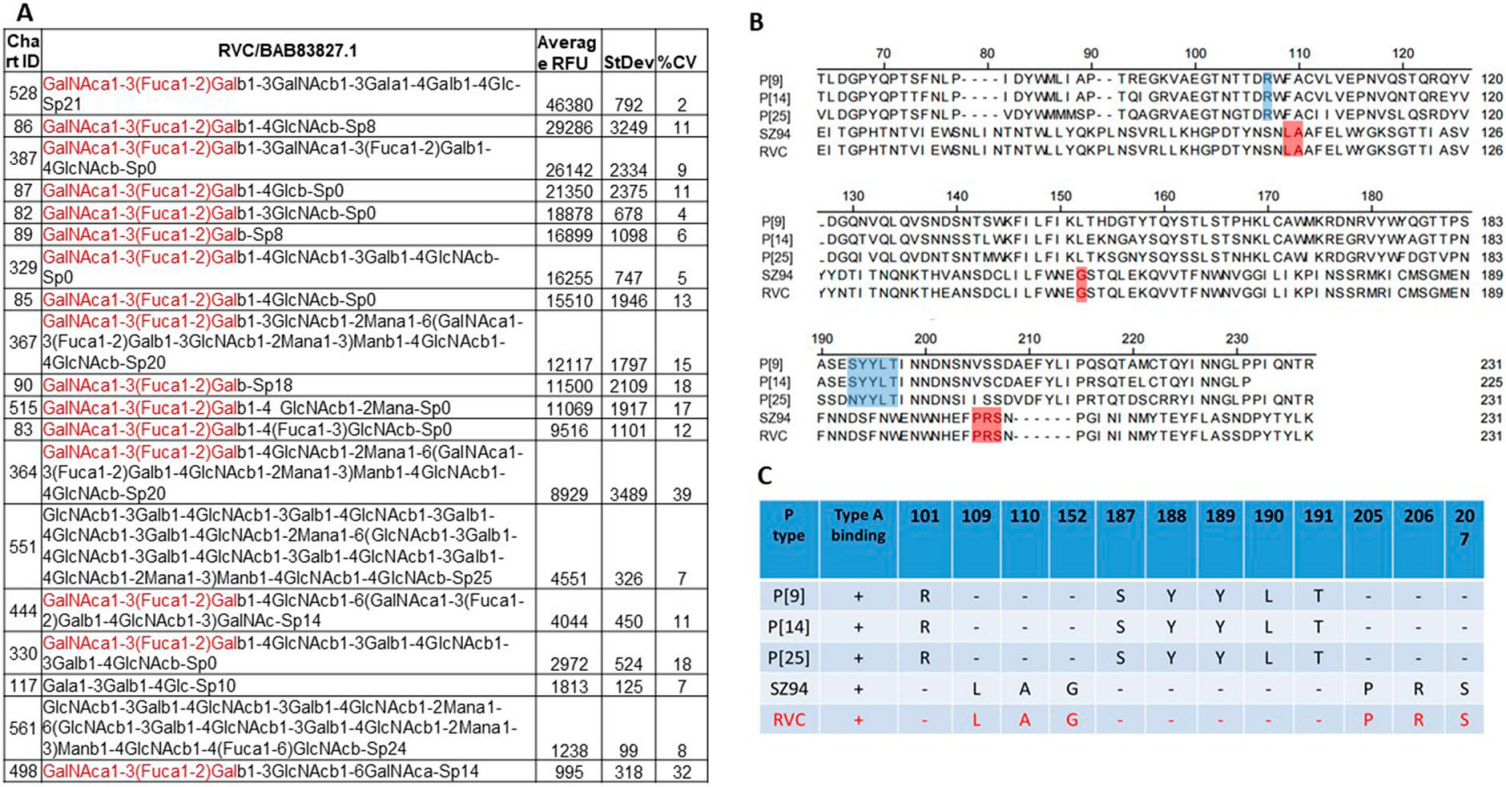
The strain representing the human RVC genogroup recognises the A antigens. Penal A showed the glycan array analyses results of the human RVC with the GalNAc-containing glycans (the A antigens) in the top list. Sequence analyses of the A antigen binding genotypes in the P[III] group RVAs with that of the human RVCs found different binding sites to the A antigens between RVAs and RVCs by the sequence alignment (Penal B) and the aligned amino acids of the binding sites (Penal C).

**The A antigen may serve as a convergent factor in host ranges and evolution between RVAs and RVCs circulating in humans and animals sharing the common A antigen.** Two RVCs were studied for glycan-binding properties by glycan array analysis. One strain (BAB83827.1) that caused several human epidemics in China [21] revealed significantly binding signals to the type A HBGAs (Figure 3). Saliva binding assays of VP8* of this RVC strain also showed a typical binding pattern to A and AB positive human saliva, consistent with the report that human RVCs recognise the A antigens [21]. In fact, the A antigen binding-human RVs also have been found in three P genotypes (P[9], P[14] and P[25]) in the P[III] genogroup of RVAs [8]. Interestingly, sequence alignment showed that the amino acid compositions of the A antigen-binding interfaces were highly conserved within but not between the two RV groups (Figure 3), suggesting an evolutionary relationship between these RVAs and RVCs by the A antigen as a convergent evolutionary factor.

**Figure 4.**
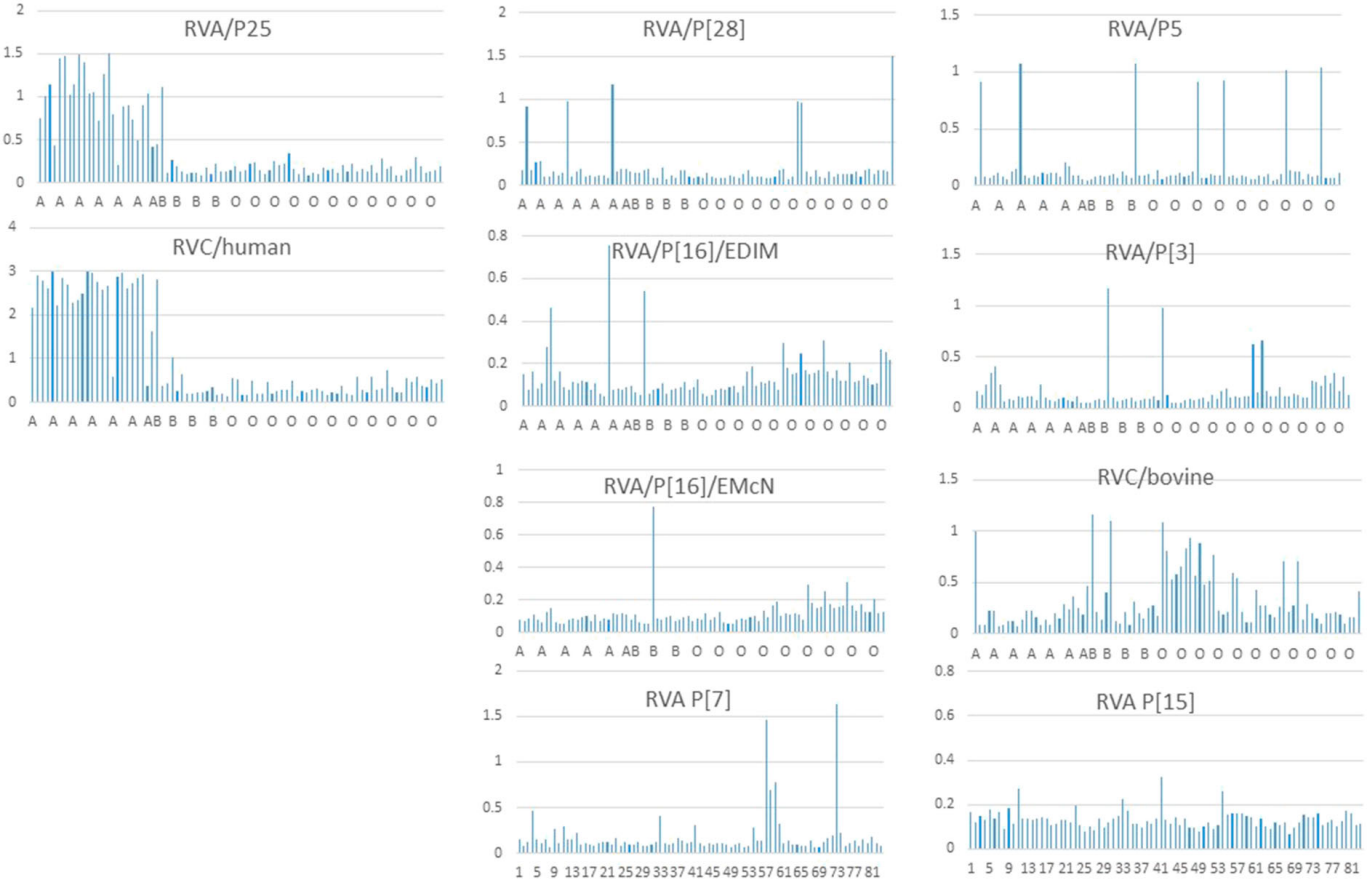
Saliva binding profiles of selected RVAs and RVCs to a penal of human saliva with different ABH types. The saliva binding signals of individual saliva samples for individual RVAs and RVCs were shown following sorting of the saliva sample penal by the ABO types of the saliva donors.

**Prediction of host ranges and species barriers between animals and humans by saliva binding assays.** Unlike the A antigen-binding results of human saliva for the human RVAs and RVCs described above, a wide diverse binding profiles of human saliva samples have been observed among strains representing eight RVA and RVC genotypes mainly infecting animals (Figure 4). These results suggested additional unidentified polymorphisms of HBGAs or glycans that exist in humans and shared with many animal species. On the other hand, the low binding rates to only small subsets of the saliva donors observed for individual genotypes suggested strong species barriers between humans and these animal RV genotypes, consistent with the fact that these genotypes mainly infect different animal species with only a few genotypes occasionally infect humans (Table 1).

## Discussion

**Figure 5.**
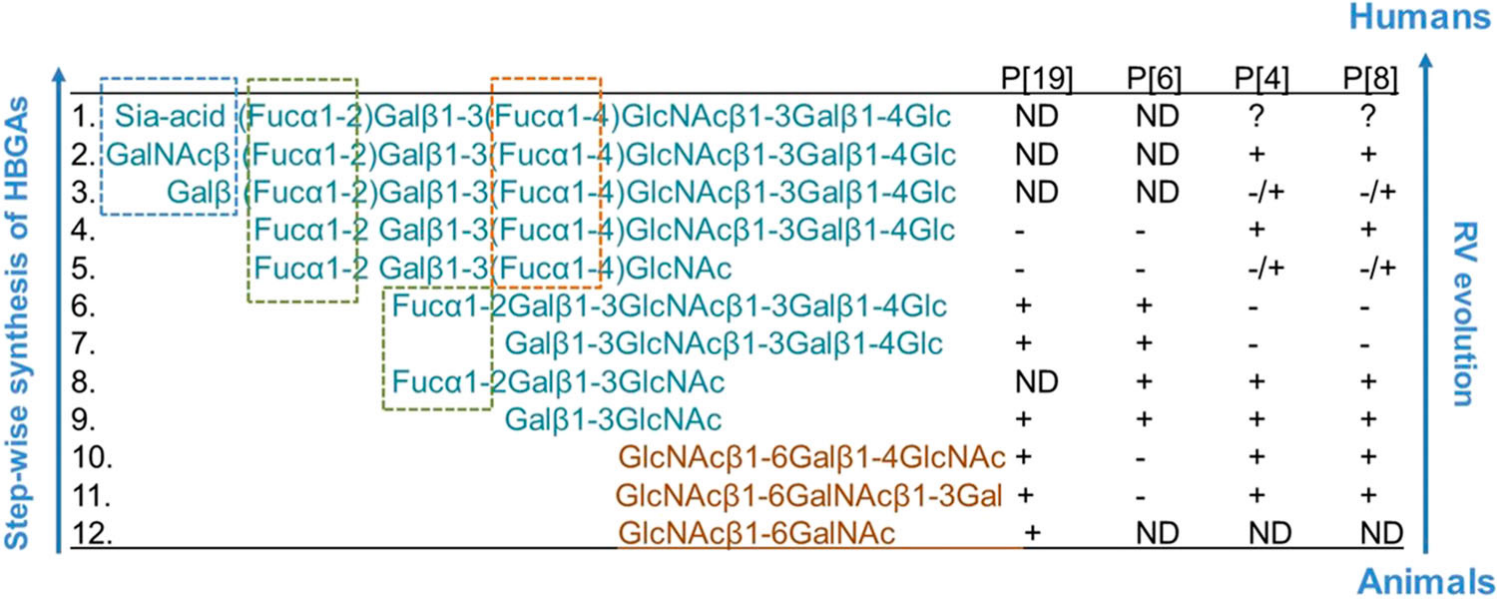
Summary of P[II] RV evolution under selection of type 1 HBGAs. The P[II] genogroup of RVs (P[4], P[6], P[8] and P[19]) that mainly infect humans has been deduced originating from an animal RV ancestor in P[I] genogroup that mainly infects different animals. P[19] represents an early stage as an intermediate jumping from animals to humans via recognising shared structures of the type 1 HBGA precursors commonly seen in humans and the mucin cores commonly seen in animals. P[6] is more advanced and recognises the type 1 precursors without the Lewis fucose (brown box) and has lost binding property to mucin cores. The P[4] and P[8] RVs are much further advanced and have broad host ranges to the secretor and Lewis positive individuals expressing the type 1 HBGAs with both Lewis and secretor fucoses, in which the Lewis fucose has been considered as a positive selection factors in P[4] and P[8] evolution. Three mucin cores are listed in brown colour while the rest type 1 HBGAs are in light blue colour. The brown dashed line box shows Lewis fucoses; the green boxes are secretor fucoses, and the blue box are sialic acid (top), the A antigen (middle), and the B antigen (bottom). The binding activities of individual P[II] RVs are listed as “-”, no binding; “+”, positive binding; “ND”, test not done; and “?”, unknown.

In our previous studies, we have demonstrated an evolutionary pathway of the P[II] RVAs that mainly infect humans responsible for ∼95% global RV epidemics. These P[II] RVAs were originated from an ancestor with a potential animal host origin in P[I] genogroup that mainly infecting different animals [14,15]. Genetic analyses also revealed a co-evolutionary relationship of P[II] RVs with humans under selection of the step-wisely synthesised H type 1 HBGAs in humans that are developmentally regulated in newborn infants and evolutionarily conserved with some animals (Figure 5) [8,14–16]. Structural/functional analyses of VP8*of P[II] RVs also elucidated the molecular details of receptor binding property changes in each step of RV co-evolution with humans reflecting the step wise synthesis of HBGAs in humans vs. some animal species, explaining the genotype-specific host ranges, disease burden, epidemiology and vaccine development against RVs [15,16].

By performing genetic and *in vitro* binding experiments of the P[I] RVAs that mainly infect different animal species, we further extended our understanding on RV evolution under selection of the step-wisely synthesised HBGAs in different animal species before they entered humans and became the major P[II] human RVs. The αGal transferase that catalyses the synthesis of the αGal glycan is found in many animal species, but it is inactivated in humans and other primate species [24]. Analysing the glycan array binding profiles of individual genotypes also identified several P[I] clusters (sub-lineages) from a common αGal-ancestor under selection of step wise synthesis HBGAs in their animal hosts before introduction to humans, further improved our understanding on RV diversity, host ranges and cross-species transmission which should facilitate future RV strain and epidemic prediction and vaccine development against RV diseases.

The A antigen-binding human RVCs described in this study represent a typical host range change from bovine to humans by switching binding properties from αGal(Galα1-3Galβ1-4GlcNAc) to the A antigens (GalNAc-Galβ1-GlcNAc) by structural adaptation from the terminal αGal to βGalNAc of the receptor binding interfaces. Both of the αGal- and the βGalNAc-glycan transferases are produced in bovine [24,28], supporting such binding specificity switch and the bovine-to-human host range changes. Similar receptor binding changes and animal-to-human host range switch has also been described in our previous studies of the three P[III] genotypes (P[9], P[14] and P[25]) of RVAs [8]. Thus, the αGal-to-GalNAc binding switch could be a common mechanism of RV evolution with host range changes under selection of stepwise synthesis of HBGAs in many animal species, explaining the P[III] RVAs commonly infect a subset of A antigen-positive humans and many antelope animals because the A enzyme is known to be produced in many antelope species [28] in addition to humans.

The terminal sialic acid residues could be another example of binding property change resulting in host ranges changes of the SA-dependent RVA genotypes of the P[I] genogroup, which has been deduced based on comparison of glycan-binding profiles of the five SA-dependent RVAs (P[3]-P[7]-P[5]-P[15]-P[23]) described in the result section (Figure 2). These data support the previous conclusion of many animal RVs recognising sialic acids based on the neuraminidase sensitive tests and the proposed co-evolution of RVs with many animal species under selection of sialic acid-containing glycan away from glycan αGal. Elucidation of such step specific host range changes is significant for disease control such as vaccine development against RVs. For example, the finding of the well adapted sialic acid binding properties of P[15] and P[5] RVAs and their low rates of human saliva binding suggests that neither P[15] lamb nor P[5] bovine RVs are suitable candidate for developing live attenuated human vaccines because they may not replicate efficiently in humans because of the species barriers.

The saliva binding assays of many animal RVs performed in this study also provide further information on the host ranges and species barrier between humans and many animal RV genotypes in the early stages of RV evolution. For example, the P[I] P[28] RVAs recognised only a small subset (5 of 81, 6%) of human saliva samples, explaining why P[28] RVs only cause sporadic cases in humans because they recognise the H type 1 HBGA precursors similar to that of the P[II] P[6] and P[19] RVAs only cause sporadic cases in young children [14,15]. Similarly, the other seven animal RVA and RVC genotypes are also at an early stage of RV evolution recognising less advanced HBGAs compared with the P[II] P[4] and P[8] RVs recognising much matured HBGAs containing the major ABH and Lewis determinants (Figure 5), explaining why these animal RV genotypes mainly infect different animal species and only occasionally infect humans.

In this study, we also included studies of RVCs to further extend our understanding of host HBGAs as an important receptor or host susceptible factor. We deduce that the same principle of co-evolution with different animal species and humans determined by their stepwise synthesis of HBGAs in parallel with RVAs. In comparison, however, the RVAs seem much more advanced than RVCs in term of recognising more mature HBGAs in humans involving the major A, H and Lewis epitopes of the human HBGAs, while RVCs recognise only the A epitope, consistent with the fact that RVAs are major human pathogens.

Our study also has limitations. For example, the assessment of the roles of αGal in RV binding and host ranges was based on comparisons of the ranks and binding signals of αGal in relative to other top-list glycans of individual RVs and the binding signals varied significantly among RV strains tested, including strains had only marginal binding signals, which need to be verified in future studies. In addition, the glycan array library used in this study contained only 610 glycan ligands which may miss important host receptor ligands. Therefore future studies with alternative glycan array libraries such as the “Mucin Array” may be used [14,29]. Furthermore, the saliva binding results revealed a widely diverse binding profiles with low binding rates to human saliva samples by many RVAs and RVCs tested, including strains exclusively infecting animals, indicating unidentified polymorphism of HBGAs in humans and shared with some animals. Future studies to verify these binding signals responsible for RV infection and replication are necessary.
